# Methicillin-resistant *Staphylococcus aureus* in Community-acquired Skin Infections

**DOI:** 10.3201/eid1106.040641

**Published:** 2005-06

**Authors:** Gregory J. Moran, Ricky N. Amii, Fredrick M. Abrahamian, David A. Talan

**Affiliations:** * Olive View–University of California at Los Angeles (UCLA) Medical Center, Sylmar, California, USA;; † David Geffen School of Medicine at UCLA, Los Angeles, California, USA

**Keywords:** cellulitis, MRSA, Staphylococcus aureus, abscess, Community associated methicillin-resistant Staphylococcus aureus, skin and soft tissue infection

## Abstract

Community-associated methicillin-resistant *Staphylococcus aureus* (MRSA) is the most common pathogen among patients with skin and soft tissue infections seeking treatment at a Los Angeles (USA) area emergency department. The proportion caused by MRSA increased from 29% in 2001 to 2002 to 64% in 2003 to 2004. No clinical or historical features reliably predict MRSA etiology.

Historically, methicillin-resistant *Staphylococcus aureus* (MRSA) infection was associated with patients in hospitals and skilled nursing facilities. In recent years, reports of community-associated MRSA infections (CA-MRSA) have been increasing (1,2). Such outbreaks have been associated with prisons, intravenous drug use, athletic teams, and men who have sex with men (1,2). CA-MRSA has primarily been described in skin and soft tissue infections (SSTIs), but the agent has also been associated with severe sepsis and pneumonia, primarily in pediatric patients (3,4). Recent studies have described an increasing proportion of MRSA isolates that are community-associated compared to hospital-associated isolates (5), but we are not aware of any published studies reporting the prevalence of CA-MRSA among patients with sporadic SSTI. The proportion of SSTIs that are caused by CA-MRSA has important implications for empiric antimicrobial therapy.

## The Study

We participated in several clinical trials of antimicrobial drugs for SSTI for which cultures were obtained from all enrolled patients. This opportunity made it possible for us to determine the prevalence of CA-MRSA among a group of emergency department patients with SSTIs. This report describes the proportion of emergency department patients with community-acquired SSTIs due to MRSA.

The study was performed in a county-owned hospital in the Los Angeles, California (USA) area, which serves a largely uninsured, low-income population. More than 43,000 persons are treated in the emergency department each year. At the hospital, we have participated in a number of clinical trials of various antimicrobial agents for treating SSTIs. All patients enrolled in these studies had cultures obtained from the infected site. Eligibility criteria for the studies included age ≥18 and an SSTI with purulent material available for culture. One study included patients with uncomplicated infections that were suitable for outpatient treatment with oral agents. Patients were also enrolled in 3 studies of complicated infections for which the treating physicians believed admission for intravenous antimicrobial drugs was indicated. Patients were excluded if they had previously received antimicrobial drugs for the infection, unless antimicrobial drugs had been taken for >72 hours with treatment failure. Patients were also excluded if they had simple abscesses that did not require antimicrobial agents, if they had severe infections involving bone or joint, or if they required amputation of an affected limb.

Specimens were obtained from the site of infection and transported by using sterile Dacron swabs. Specimens were processed and cultured with standard techniques (6). *S. aureus* was identified by colony morphologic features, coagulase tests, and catalase tests. MICs were determined by VITEK, GPS 106 or 109 card (bioMérieux, Durham, NC, USA), according to manufacturer's instructions. MIC breakpoints and quality control protocols were used according to standards established by NCCLS (7).

Clinical data were prospectively collected as part of the clinical trials. In mid-2002, we began prospectively collecting information on recent jail exposure. Those patients enrolled previously were contacted by telephone, if possible, to obtain information on jail exposure. This study was approved by the Olive View–UCLA institutional review board.

From January 2002 through December 2002, a total of 24 patients were enrolled in an outpatient antimicrobial drug study. From August 2001 through March 2004, we enrolled 72 patients in 3 inpatient studies, and each had only 1 site of infection. Patients were 20–60 years of age, with a median age of 42. Men made up 77% of the study group. None of the patients resided in long-term care facilities, and none of the infections was believed to be hospital-acquired.

MRSA was isolated from 44 (46%) of 96 patients (8 outpatients, 36 admitted). The proportion of infections yielding MRSA increased from 14 (29%) of 49 during 2001 to 2002 to 30 (64%) of 47 from January 2003 through March 2004. Other pathogens isolated included the following: 15 methicillin-susceptible *S. aureus*, 19 *Streptococcus* spp., 4 coagulase-negative staphylococci, 2 diphtheroids; 2 *Citrobacter* spp., 2 *Escherichia coli*; and 1 *Enterococcus* sp. No organism was isolated from 7 patients.

Among 44 MRSA patients, 6 had been hospitalized within the last year. None had indwelling catheters or other recognized risk factors for MRSA. Five had diabetes; otherwise, none had a notable associated coexisting illness. Fifteen had previously received oral antimicrobial drugs for the current infection, but treatment was unsuccessful. Nine had recently used injected illegal drugs. Nine were homeless. Of 36 MRSA patients for whom the information was available, 3 had been in Los Angeles County Jail within the last year, where an MRSA outbreak was recently described (2). Most patients had no apparent epidemiologic risk factors associated with recent CA-MRSA outbreaks. No clinical or epidemiologic features were predictive of an MRSA cause (Table).

**Table:** Clinical features and epidemiologic characteristics of patients with skin and soft tissue infections*

Feature/characteristic	MRSA, n = 44 (%)	Other pathogens, n = 52 (%)
Hospitalized in last year	6 (14)	6 (12)
Prior, unsuccessful antimicrobial drug treatment	15 (34)	15 (29)
Injection drug use	9 (20)	16 (31)
Jail in last year†	3/36 (8)	3/48 (6)
Homeless	9 (20)	10 (19)
Abscess present	41 (93)	44 (85)

*MRSA, methicillin-resistant *Staphylococcus aureus*. †This information was not available for 8 MRSA patients and 4 patients with other pathogens.

Antimicrobial susceptibilities of the 44 MRSA isolates were as follows: clindamycin 98%, erythromycin 2%, levofloxacin 16% (64% had intermediate susceptibility to levofloxacin), rifampin 98%, tetracycline 82%, trimethoprim/sulfamethoxazole 100%. Fourteen of our MRSA isolates from early 2003 were tested in the laboratory at the Los Angeles County Department of Health Services. The isolates were found by pulsed-field gel electrophoresis to be identical to the strain associated with the outbreak at the Los Angeles County Jail, which belongs to the USA 300 ST:8 group (8,9). None of the 14 isolates tested had inducible clindamycin resistance by the D test.

## Conclusions

Our report demonstrates that the proportion of patients with community-acquired SSTI caused by MRSA is increasing, and CA-MRSA is now the most common cause of community-acquired SSTIs at our center. Other reports have suggested that CA-MRSA is becoming more common in other geographic areas in the United States and Europe (10,11). A high proportion of CA-MRSA strains (such as the USA 300 ST:8 strain) have been found to carry the Panton-Valentine leukocidin gene, which has been associated with SSTI and necrotizing pneumonia (9,12). We have noted anecdotally that many patients with CA-MRSA exhibit a spontaneous abscess or furunculosis that the patient thinks was caused by a spider bite.

The bacterial causes of common community-acquired SSTIs are generally gram-positive organisms such as *S. aureus* and *Streptococcus pyogenes*. Because of the predictable etiology of these infections, most physicians do not routinely obtain cultures from these patients. Obtaining cultures of SSTIs is now of greater importance to monitor the extent of CA-MRSA infections in one's community and guide therapy in areas in which CA-MRSA is already prevalent.

Most community-acquired SSTIs are treated with antimicrobial drugs such as cephalexin and dicloxacillin. Patients requiring intravenous therapy are most commonly given agents such as cefazolin or oxacillin. In areas with a high prevalence of CA-MRSA, empiric treatment for SSTIs with β-lactam agents such as cephalexin or dicloxacillin may no longer be appropriate. Oral agents such as clindamycin or trimethoprim/sulfamethoxazole and rifampin should be considered for CA-MRSA. Although inducible clindamycin resistance was not found in the few patients we tested, clinical failure due to inducible clindamycin resistance among CA-MRSA has been reported (13). Whether the addition of rifampin to trimethoprim/sulfamethoxazole improves outcomes in SSTI is not clear, but this combination appears to be more effective in eradicating MRSA colonization (14). Macrolides, tetracycline, and fluoroquinolones have inconsistent activity against the MRSA isolates identified in our study and other reports of CA-MRSA (11). For severe infections treated in the inpatient setting, clindamycin or vancomycin should be included as part of empiric therapy.

Adequate drainage and débridement of SSTIs are important in treatment. We did not find a higher rate of MRSA among those patients in whom previous antimicrobial drug treatment had been unsuccessful and believe inadequate drainage was the reason.

Whether additional measures to eliminate carriage of MRSA in these patients or their close household contacts would be of any benefit is not known. Chlorhexidine body washes and nasal mupirocin would be reasonable measures for those with recurrent SSTI or close contacts with similar infections (15).

Our report has several limitations. One of the criteria for study enrollment was availability of purulent material for culture. Most patients had skin abscesses. Patients with cellulitis without a purulent exudate are not represented in our study sample. We did not culture every possible SSTI seen at the emergency department, but we believe the patients enrolled in these studies reflect the general population with culturable SSTIs. All samples cultured during 2003 to 2004 were from patients with infections that required hospital admission, so these results may not reflect those patients with minor infections suitable for outpatient treatment. Prevalence of CA-MRSA can vary considerably between geographic areas, and our facility may not be typical of southern California or other areas.

MRSA may now be the most common pathogen among patients with community-associated SSTIs in some areas. Physicians should consider obtaining cultures in these patients. In areas with a high prevalence of CA-MRSA, empiric therapy for SSTIs with agents such as clindamycin or trimethoprim/sulfamethoxazole and rifampin would be appropriate.
